# Assessment of Pain During Pediatric Dental Treatment Using Different Sedative Agents: A Crossover Trial

**DOI:** 10.7759/cureus.41676

**Published:** 2023-07-11

**Authors:** Palak Janiani, Deepa Gurunathan, Ramsesh Manohar

**Affiliations:** 1 Department of Pediatric and Preventive Dentistry, Saveetha Dental College and Hospital, Saveetha Institute of Medical and Technical Sciences, Saveetha University, Chennai, IND; 2 Department of Anaesthesiology, Saveetha Dental College and Hospital, Saveetha Institute of Medical and Technical Sciences, Saveetha University, Chennai, IND

**Keywords:** clinical pediatric dentistry, dexmedetomidine, midazolam, inhaled nitrous oxide, intranasal administration

## Abstract

Background

Behavioral management techniques are employed for children who are fearful and uncooperative. Pharmacologic sedation and anesthesia are frequently utilized to manage pain and anxiety in pediatric dental patients.

Aim

To evaluate the intraoperative and postoperative pain levels during dental treatment of children sedated with 1.5 μg/kg intranasal dexmedetomidine, 0.3 mg/kg intranasal midazolam, and nitrous oxide.

Materials and methods

In this crossover study, 24 children between the ages of five and seven years were randomly assigned to receive intranasal atomized dexmedetomidine, intranasal atomized midazolam, and inhaled nitrous oxide during three different visits. At each visit, a single pulp therapy procedure was conducted after administering the respective sedative agent, and the pain levels were documented. There was a one-week interval between each visit to allow for a washout period. The data were analyzed using IBM SPSS Statistics for Windows, Version 22.0 (Released 2013; IBM Corp, Armonk, NY, United States) using the Wilcoxon signed-rank test and Kruskal-Wallis H test (p < 0.05).

Results

All three sedative agents were equally effective in controlling postoperative and intraoperative pain. Although there was no statistically significant difference among the groups, clinically, midazolam showed lower intraoperative pain levels (mean 1.78 ± 1.42).

Conclusion

In pediatric dental patients, intranasal midazolam at a dosage of 0.3 mg/kg and intranasal dexmedetomidine at a dosage of 1.5 μg/kg demonstrate comparable effectiveness to nitrous oxide sedation in pain management. These options serve as effective alternatives for anxious children who may not tolerate nitrous oxide sedation.

## Introduction

Pharmacological techniques, such as sedation, are utilized to manage children's behavior during dental procedures when non-pharmacological methods prove ineffective. Conscious sedation is often favored over general anesthesia due to its lower cost and decreased risk, but its effectiveness depends on the specific medication and delivery method employed [1]. Various routes of drug administration are employed, including oral, nasal, intravenous, and inhalation methods, each with its own advantages and disadvantages [2]. The intranasal route is gaining popularity due to its quicker onset of action and relatively simple administration technique.

Sedative agents such as midazolam, ketamine, dexmedetomidine, and sufentanil are commonly administered through the intranasal route [3]. Dexmedetomidine is considered the safest choice for children and infants since it has minimal impact on respiratory function and does not interfere with the heart rate [4]. Conversely, midazolam is seen as an excellent sedative agent due to its favorable balance between toxicity and effectiveness, a wide range of therapeutic doses, a safety margin, and a relatively short duration of action. These characteristics contribute to its ability to induce sedation quickly and promote swift recovery when compared to other agents [5].

The Council of European Dentists endorses nitrous oxide-oxygen sedation, commonly referred to as "laughing gas," as the recommended standard method of sedation [6]. It provides quick onset and recovery, the ability to adjust the dosage, and minimal risks to the systems of the body. However, successfully administering nitrous oxide relies on proper technique and the patient's willingness to accept the mask, which can be difficult for children with high anxiety levels [7].

In order to overcome the drawbacks associated with nitrous oxide-oxygen sedation and capitalize on the benefits of intranasal dexmedetomidine and midazolam, a comparative study was carried out to evaluate the levels of pain experienced during and after dental procedures in children who were sedated with atomized intranasal dexmedetomidine (1.5 μg/kg), atomized intranasal midazolam (0.3 mg/kg), and nitrous oxide-oxygen sedation. The null hypothesis states that there is no difference in the intraoperative and postoperative pain levels during dental procedures in children who were sedated with atomized intranasal dexmedetomidine (1.5 μg/kg), atomized intranasal midazolam (0.3 mg/kg), and nitrous oxide-oxygen sedation.

## Materials and methods

Study design and protocol

This clinical trial was a randomized, three-armed crossover study that commenced after obtaining ethical clearance from an institution (IHEC/SDC/PEDO-2001/21/334). To detect any discrepancies between the groups, the study required a sample size of 20 participants with 85% statistical power at a significance level of 0.05. To accommodate a potential loss to follow-up of up to 20%, an additional four children were included, resulting in a total of 24 participants. Eligible children were between the ages of five and seven years, exhibiting "negative" behavior according to the Frankl scale and having an American Society of Anesthesiologists I (ASA I) physical status, who required pulp therapy in at least three lower molars. Exclusion criteria included children with known hypersensitivity to benzodiazepines, any systemic disease, special needs, or any condition that increased the risk of airway obstruction. 

The children were randomly divided following a computer-generated sequence of random numbers. The allocation was done using opaque, sealed brown envelopes. The study protocol, including the treatment's associated risks and benefits, was meticulously communicated to the parents following which informed consent was obtained. Before inclusion, attempts were made to implement basic behavior modification techniques, and only children who did not respond to these techniques were included in the study. Additionally, a comprehensive evaluation of the general health of all patients was carried out by the anesthetist at the hospital prior to their enrollment. The fasting protocol, as outlined by the American Academy of Pediatric Dentistry, was diligently followed before every visit [2]. On the day of treatment, the child underwent a reassessment by the anesthetist.

Procedure

A mucosal atomization device (Wolf Tory Medical, Salt Lake City, UT, United States) attached to a 1 ml syringe was used to administer either 1.5 μg/kg of dexmedetomidine (Dextomid, Neon Laboratories Ltd, Mumbai, India) or 0.3 mg/kg of midazolam hydrochloride (Mezolam 5 g/mI, Neon Laboratories Ltd, Mumbai, India) intranasally to each patient. The sedation levels were monitored to assess their effectiveness. To provide analgesia, a relative analgesia machine (Consed, Kerala, India) was utilized, delivering a mixture of 30% nitrous oxide and 70% oxygen. The flow rate was determined by initiating the administration with 100% oxygen for two to three minutes. Following the procedure, the patients received 100% oxygen for five minutes.

**Figure 1:**
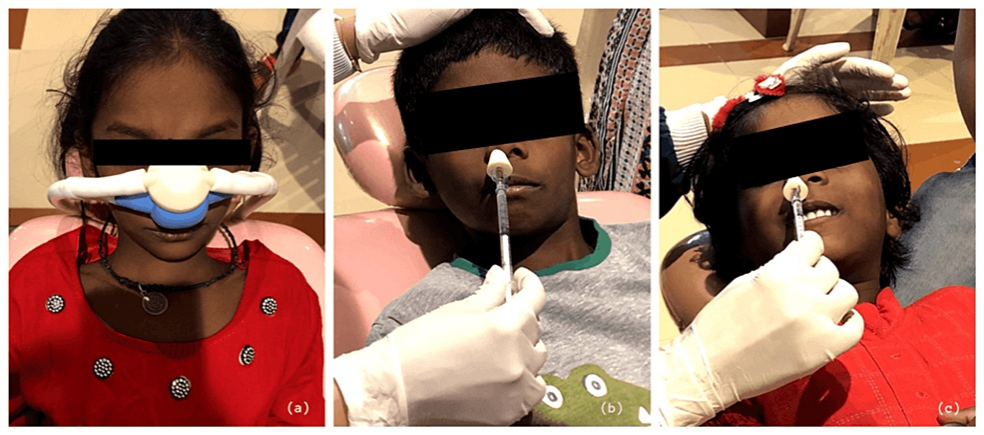
Routes of administration of sedative agents utilized in the study. (a) Nitrous oxide-oxygen sedation using a nasal mask. (b) Intranasal midazolam sedation using a mucosal atomization device. (c) Intranasal dexmedetomidine sedation using a mucosal atomization device.

Once the child demonstrated signs of relaxation, topical anesthesia was sprayed onto the injection site, followed by the administration of local anesthesia. Local infiltration of articaine with adrenaline 1:100,000 (Septanest) was performed near the specific tooth of interest before proceeding with pulp therapy. The child was continuously monitored by the anesthetist throughout the entire dental procedure. This treatment protocol was consistent across all arms of the trial, with a one-week washout period between visits. Since the methods of inducing dexmedetomidine and midazolam differ from those of nitrous oxide and can be easily distinguished, both the operator and evaluator were unable to be blinded (Figure 1).

Outcome assessment

The subjective pain levels were assessed by obtaining preoperative and postoperative pain scores using the Wong-Baker Faces Pain Scale. The children were asked to rate their pain while they waited in the sitting area and then again after the treatment was completed. Intraoperative objective pain was assessed using the Face, Legs, Activity, Cry, and Consolability (FLACC) score during the administration of local anesthesia.

Statistical analysis

Descriptive statistics were conducted using IBM SPSS Statistics for Windows, Version 22.0 (Released 2013; IBM Corp, Armonk, New York, United States). The data normality of pain scores was assessed using the Shapiro-Wilk test, which indicated that the scores followed a skewed distribution. To compare intraoperative pain scores, the Kruskal-Wallis H test was utilized. Intragroup comparisons of subjective pain before and after treatment were made using the Wilcoxon signed-rank test. A significance level of p < 0.05 was considered statistically significant for all analyses.

## Results

**Figure 2:**
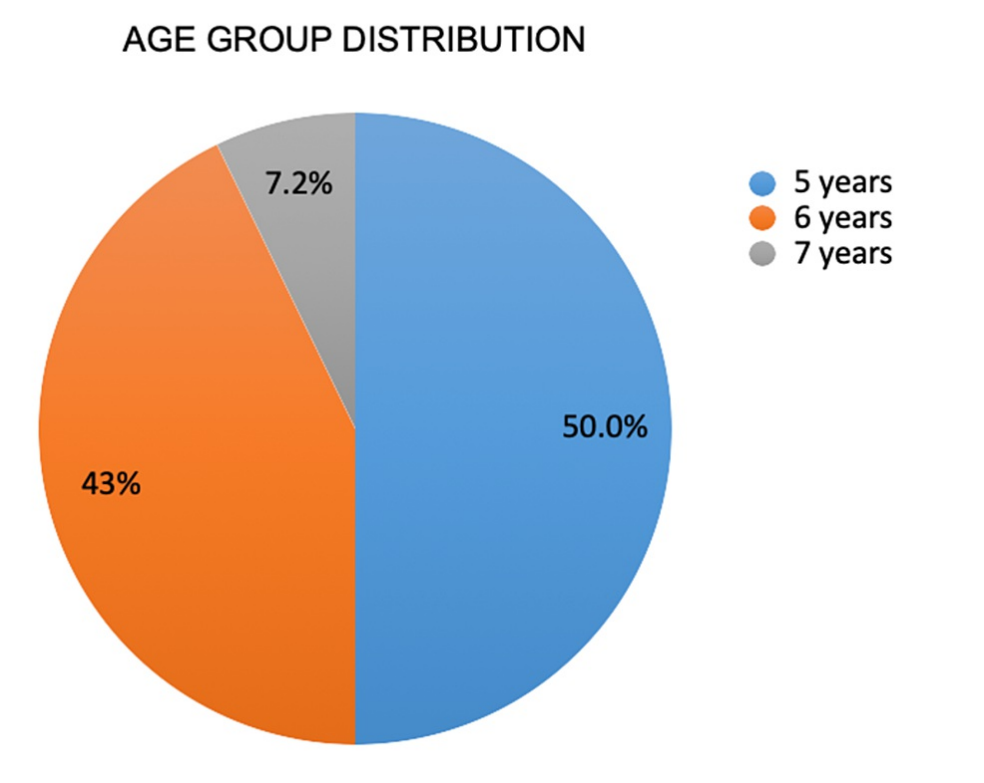
Age distribution of the included children.

The present study recruited a total of 24 children, of which 14 were female and 10 were male. However, two children did not report for all arms of the study and were therefore excluded from the analysis. Table 1 and Figure 2 present the demographic characteristics and the age distribution of the included children, respectively.

**Table 1:** Demographic characteristics of the included children.

Demographic characteristics	
Mean age (years)	5.86 ± 0.94
Sex N (%)	
Male	9 (40.9%)
Female	13 (59.1%)

**Figure 3:**
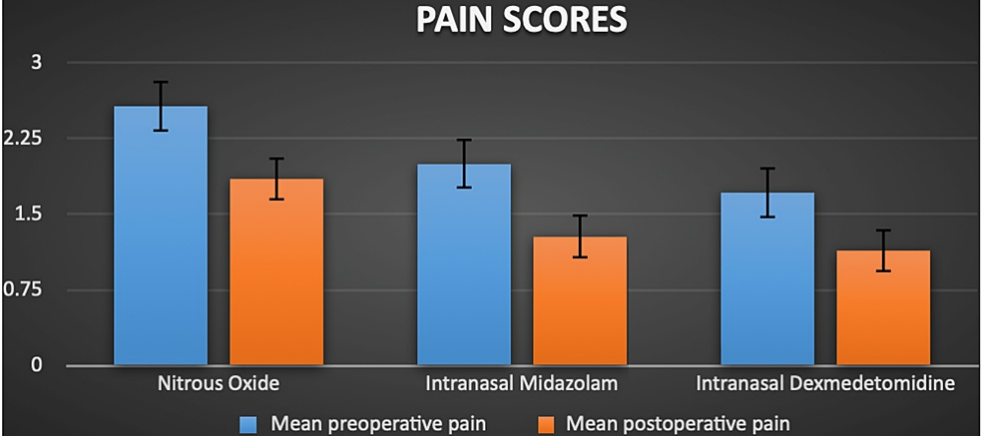
Preoperative and postoperative subjective pain scores as assessed by the Wong-Baker Faces Pain Scale.

The intragroup analysis showed that there was no statistically significant difference in the preoperative and postoperative pain scores in any of the groups (p > 0.05) (Table 2). Figure 3 depicts the mean preoperative and postoperative subjective pain scores in the three groups.

**Table 2:** Intergoup and intragroup comparison of mean preoperative and postoperative pain scores as per the Wong-Baker Faces Pain Scale. ^#^No significant difference (p > 0.05).

Wong-Baker Faces Pain Scale	Mean pain scores ± SD			Intergroup comparison p-value
	Nitrous oxide	Intranasal midazolam	Intranasal dexmedetomidine	
Preoperative	2.57 ± 2.97	2.0 ± 2.39	1.71 ± 1.97	0.830^#^
Postoperative	1.85 ± 2.37	1.28 ± 2.68	1.14 ± 1.64	0.375^#^
Intragroup comparison p-value	0.168^#^	0.263^#^	0.279^​​​​​​​#^	-

Intraoperatively, the intranasal midazolam group showed a lower mean pain score when compared to the nitrous oxide and dexmedetomidine group. However, this was found to be statistically insignificant (Table 3).

**Table 3:** Intergroup comparison of intraoperative pain (FLACC score). FLACC: Face, Legs, Activity, Cry, and Consolability. ^#^No significant difference (p > 0.05).

FLACC score	Mean FLACC score ± SD			Kruskal-Wallis H test	p-value
	Nitrous oxide	Intranasal midazolam	Intranasal dexmedetomidine		
	2.28 ± 2.46	1.78 ± 1.42	2.48 ± 1.85	0.414	0.813^#^

## Discussion

The current study is a randomized clinical trial that compares the postoperative and intraoperative pain levels during pediatric dental treatment under the influence of intranasal midazolam, intranasal dexmedetomidine, and nitrous oxide. Our findings reveal that there is no statistically significant difference among the three groups.

Midazolam, when administered orally, has limitations such as its bitter taste and variable sedative effects due to insufficient drug ingestion [8]. Therefore, the intranasal route of administration was chosen for this study. The use of a mucosal atomization device allows for better drug absorption by producing small particles of 30 μm and preventing drug leakage [9]. Based on previous research, a dose of 0.2 mg/kg of midazolam was found to be as effective as 0.3 mg/kg when combined with nitrous oxide [10]. However, since the current study did not utilize other sedative agents, a higher dose of 0.3 mg/kg was chosen.

All three sedative agents used in the study possess analgesic properties, which may explain the lack of significant variation in intraoperative pain scores. Additionally, consistent application of topical anesthetic prior to administering articaine may have contributed to the uniformity in intraoperative pain levels. Individual patient factors, including anxiety levels, pain threshold, and response to sedation, can also influence perceived pain during dental procedures [11].

Intranasal midazolam acts on the central nervous system by enhancing the effects of the inhibitory neurotransmitter gamma-aminobutyric acid (GABA). This mechanism of action produces sedative, anxiolytic, and muscle relaxant effects, which collectively contribute to pain reduction [12]. Nitrous oxide, on the other hand, exerts its analgesic properties by interacting with opioid receptors, modulating pain transmission. However, the analgesic effect of nitrous oxide is relatively weaker compared to intranasal midazolam, potentially explaining the observed differences in pain levels [13]. Dexmedetomidine exerts its analgesic effects through its action on α2-adrenergic receptors located in the central nervous system. As a selective α2-adrenergic agonist, it primarily acts on the α2A subtype receptors. Upon binding to these receptors, dexmedetomidine inhibits the release of norepinephrine, leading to the suppression of pain signaling pathways [14].

Contrary to our findings, Takkar et al. observed a notable decrease in pain scores when administering an inferior alveolar nerve block with nitrous oxide-oxygen sedation [15]. Similarly, Srinivasan et al. conducted a comparison between nitrous oxide inhalation and intranasal midazolam, concluding that intraoperative pain was significantly lower in the nitrous oxide group compared to the midazolam group [11]. In contrast, our study yielded contrasting results when compared to both of these studies.

The strength of this study lies in its design as a three-arm crossover trial, minimizing intersubject variability, and the standardized treatment protocol for each visit to reduce bias. However, limitations included longer treatment times due to the required one-week washout period between visits, potentially introducing a carryover effect. Further research is needed to explore the underlying mechanisms and patient-specific factors that contribute to the differential efficacy of intranasal midazolam, dexmedetomidine, and nitrous oxide sedation in pain management during pediatric dental treatment.

## Conclusions

In the realm of pain management for pediatric dental treatment, the use of intranasal midazolam at a dosage of 0.3 mg/kg and intranasal dexmedetomidine at a dosage of 1.5 μg/kg has demonstrated efficacy comparable to the established gold standard of nitrous oxide sedation. By employing intranasal midazolam and dexmedetomidine, dental practitioners can provide effective pain management while minimizing the potential challenges associated with nitrous oxide inhalation. These alternative methods can be especially advantageous for children who experience anxiety and are unwilling to wear the nasal mask used with nitrous oxide administration.

## References

[1] Rosenberg M (2012). Pediatric sedation outside of the operating room. Anesth Prog.

[2] Coté CJ, Wilson S, American Academy of Pediatric Dentistry, American Academy of Pediatrics (2019). Guidelines for monitoring and management of pediatric patients before, during, and after sedation for diagnostic and therapeutic procedures. Pediatr Dent.

[3] Preethy NA, Somasundaram S (2021). Sedative and behavioral effects of intranasal midazolam in comparison with other administrative routes in children undergoing dental treatment - a systematic review. Contemp Clin Dent.

[4] Mohite V, Baliga S, Thosar N, Rathi N (2019). Role of dexmedetomidine in pediatric dental sedation. J Dent Anesth Pain Med.

[5] Papineni A, Lourenço-Matharu L, Ashley PF (2014). Safety of oral midazolam sedation use in paediatric dentistry: a review. Int J Paediatr Dent.

[6] Council of European Dentists Council of European Dentists. CED Resolution on the Use of Nitrous Oxide Inhalation Sedation - Update. https://www.omd.pt/content/uploads/2019/12/CED-DOC-2019-055-E.pdf.

[7] Musani IE, Chandan NV (2015). A comparison of the sedative effect of oral versus nasal midazolam combined with nitrous oxide in uncooperative children. Eur Arch Paediatr Dent.

[8] Vallogini G, Festa P, Matarazzo G, Gentile T, Garret-Bernardin A, Zanette G, Galeotti A (2022). Conscious sedation in dentistry for the management of pediatric patients with autism: a narrative review of the literature. Children (Basel).

[9] Primosch RE, Guelmann M (2005). Comparison of drops versus spray administration of intranasal midazolam in two- and three-year-old children for dental sedation. Pediatr Dent.

[10] Fuks AB, Kaufman E, Ram D, Hovav S, Shapira J (1994). Assessment of two doses of intranasal midazolam for sedation of young pediatric dental patients. Pediatr Dent.

[11] Srinivasan NK, Karunagaran P, Panchal V, Subramanian E (2021). Comparison of the sedative effect of inhaled nitrous oxide and intranasal midazolam in behavior management and pain perception of pediatric patients: a split-mouth randomized controlled clinical trial. Int J Clin Pediatr Dent.

[12] Goswami M, Sangal A, Rahman B, Chawla S (2021). Comparison of the safety and efficacy of dexmedetomidine with midazolam for the management of paediatric dental patients: a systematic review. J Indian Soc Pedod Prev Dent.

[13] Kotian N, Subramanian EMG, Jeevanandan G (2022). Comparing the sedative effect of oral and intranasal midazolam and their effect on behavior in pediatric dental patients. Int J Clin Pediatr Dent.

[14] Zhao Y, He J, Yu N, Jia C, Wang S (2020). Mechanisms of dexmedetomidine in neuropathic pain. Front Neurosci.

[15] Takkar D, Rao A, Shenoy R, Rao A, Saranya BS (2015). Evaluation of nitrous oxide inhalation sedation during inferior alveolar block administration in children aged 7-10 years: a randomized control trial. J Indian Soc Pedod Prev Dent.

